# Structural Analysis of Cu^+^ and Cu^2+^ Ions in Zeolite as a Nanoreactor with Antibacterial Applications

**DOI:** 10.1021/acsomega.3c03869

**Published:** 2023-08-08

**Authors:** J. Flores-Valenzuela, J. E. Leal-Perez, J. L. Almaral-Sanchez, A. Hurtado-Macias, A. Borquez-Mendivil, R. A. Vargas-Ortiz, B. A. Garcia-Grajeda, S. A. Duran-Perez, Manuel Cortez-Valadez

**Affiliations:** † Universidad Autónoma de Sinaloa, Fuente de Poseidón y Prol. Ángel Flores S/N, Los Mochis, Sinaloa 81223, México; ‡ Centro de Investigación en Materiales Avanzados, S. C., Miguel de Cervantes #120, Complejo Industrial Chihuahua, Chihuahua, Chihuahua 31136, México; § Doctorado en Biotecnología, Facultad de Ciencias Químico Biológicas, Universidad Autónoma de Sinaloa, Calzada de las Americas Norte #2771, Burócrata, Culiacán Rosales, Sinaloa 80030, México; ∥ Departamento de Investigación en Física, Universidad de Sonora, Apdo. Postal 5-88, Hermosillo, Sonora 83190, México

## Abstract

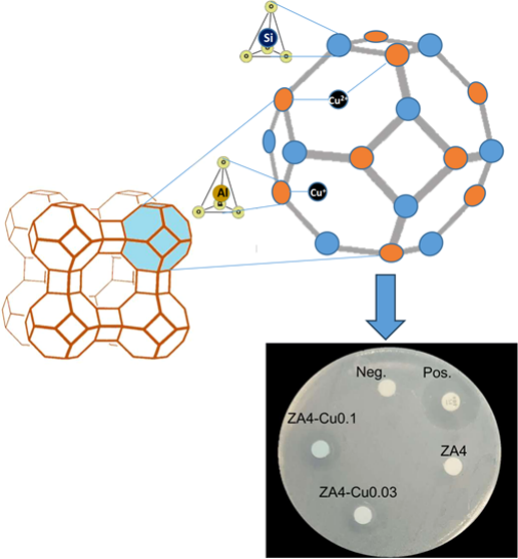

In this work, we report the structural analysis of Cu^+^ and Cu^2+^ ions in zeolite as a nanoreactor with antibacterial applications. A simple one-step process was implemented to obtain Cu ions in zeolite A (ZA4) by controlling the temperature in the solutions to guarantee the ions’ stability. Samples were characterized by scanning electron microscopy, energy-dispersive X-ray spectroscopy, and Fourier transform infrared (FT-IR) spectroscopy, showing the characteristic zeolite elements as well as the characteristic bands with slight modifications in the chemical environment of the zeolite nanoreactor attributed to Cu ions by FT-IR spectroscopy. In addition, a shift of the characteristic peaks of ZA4 in X-ray diffraction was observed as well as a decrease in relative peak intensity. On the other hand, the antibacterial activity of Cu ions in the zeolite nanoreactor was evaluated.

## Introduction

Compared to natural zeolites, synthetic zeolites like A, X, ZSM-5, Y, P, and Na-P1 offer significant benefits due to their wider range of industrial applications. These include serving as cation exchangers, catalytic supports, selective adsorbents, and catalysts.^1−6^ Due to their ability to facilitate chemical reactions in a controlled environment, zeolites fulfill the criteria to be classified as nanoreactors.^7,8^ Moreover, the zeolite nanoreactor has the potential to generate a distinct and exclusive chemical environment by isolating compounds and influencing the reaction within the nanoreactor.^9^ It can also be utilized to facilitate the creation of uniformly sized nanoparticles and prevent undesirable nanoparticles from sintering.^10^ Zeolite has oxide-reduction capacity, which can produce Cu^2+^, Cu_,_^+^ and metallic Cu,^11,12^ including clusters of Cu^3+^.^13^ Studies have been carried out on the ion exchange of Cu^2+^ in zeolites ZSM-5, Y, CHA, X, RTH, and LTA^14−23^ and Cu^+^ has been studied in ZSM-5, CHA, and BEA^24−26^ independently. A recent investigation of copper mono- and polystructures obtained in a ZSM-5 zeolite has revealed how the copper species interact with the zeolite, as well as precisely observing their positions within the zeolite structure.^27^ For ZA4, there are four active sites for Cu^2+^: SII, which is in the center of a six-ring face; SII′, located in the center of the sodalite, SII*, in the large cage; and SIII, in the D4R.^28^ Moreover, studies have revealed that the redistribution in electron density modifies the distribution of charge in the Al–O bonds when the coordination of metal cations occurs in cation exchange sites in zeolites.^29^ The selectivity of ion exchange generally increases as the charge and size of the exchanger ion increase.^30^ Additionally, zeolites have demonstrated antibacterial properties, such as natural zeolite tuffs that contain Cu^2+^, Zn^2+^, or Ni^2+^, and have been found to be highly effective against *Escherichia coli* and *Staphylococcus aureus*.^31^ In experiments involving zeolite X and A with different Al/Si ratios and which were ion-exchanged with Ag^+^, Zn^2+^, and Cu^2+^, it was discovered that Ag^+^-ion-loaded zeolites exhibited the most significant antibacterial activity compared to other metal-ion-embedded zeolites.^32^ Another study reports that the Cu^2+^-ZnO-modified 13X zeolite has excellent antibacterial activity against *E. coli* and *S. aureus*.^33^ Infections caused by bacteria like *E. coli* and *S. aureus* are the main causes of bacteremia and healthcare-associated infections (HAIs). Also, it is getting harder to treat infections because some bacteria have become stronger than others. Therefore, research on zeolites as a means to reduce antibacterial activity has revealed a high potential due to their large surface area and capacity to exchange metal cations, which have been extensively demonstrated for the elimination of these microorganisms, as shown in other investigations.^34−37^ However, no published work has been found on Cu^+^ or Cu^2+^ ion analysis in ZA4 on structural and antimicrobial activity simultaneously. For this reason, we are interested in understanding if the addition of Cu ions to Z4A confers a potential antimicrobial agent. At present, only previous works on the alteration of the crystal structure of Z4A, attributed to the presence of Cu^2+^ ions,^38^ have been found; however, they only studied the adsorption and removal of Cu(II). The purpose of this study was to determine the impact of Cu^+^ and Cu^2+^ ions in ZA4 and their effect on structural and antibacterial activity. The samples were analyzed by scanning electron microscopy-energy dispersive X-ray spectroscopy (SEM-EDS) to show the morphology and chemical composition of the samples, Fourier transform infrared (FT-IR) spectroscopy to identify changes in the chemical environment of the molecular structure of zeolite, and X-ray diffraction (XRD) to analyze changes in the crystal structure of the zeolite that vacancies or atomic substitutions can cause.

## Experimental Section

### Materials

Synthetic zeolite 4A (ZA4, Sigma-Aldrich), CuSO_4_·5H_2_O (99.5%, Sigma-Aldrich), deionized water, Gram-positive bacteria *S. aureus* (ATCC BAA-1026), and *Enterococcus faecalis* (ATCC 29212).

### Methods

#### Cu Ion Exchange

10 g of Z4A in 25 mL of deionized water was prepared for 24 h by hydration. Independently, 100 mL solutions of CuSO_4_ for two solutions with 0.03 M (Z4A-Cu0.03) and 0.1 M (Z4A-Cu0.1) were elaborated. Subsequently, the CuSO_4_ and hydrated zeolite solutions were placed independently in a thermal bath until obtaining a constant at 50 °C. Then, under the same temperature conditions, each CuSO_4_ solution was added to the hydrated zeolite and maintained under magnetic stirring for 25 min to form Z4A-Cu0.03 and Z4A-Cu0.1. The solution was filtered and washed three times with deionized water to remove the remaining ions. Subsequently, drying was carried out at room temperature (30 °C in summer) to preserve the stability of the Cu ions.

#### Antibacterial Activity Test

The antibacterial activity of Z4A, Z4A-Cu0.03, and Z4A-Cu0.1 was tested against the Gram-positive bacteria *S. aureus* (ATCC BAA-1026) and *E. faecalis* (ATCC 29212). The bacteria were pre-grown on Luria Bertani (LB) medium overnight at 37 °C to obtain the cultures in the log phase of growth. The bacteria were diluted to 10^6^ CFU using phosphate-buffered saline (PBS) and plated on Mueller–Hinton agar plates. 6 mm diameter Whatman filter paper disk were impregnated with the different materials at a concentration of 0.1 g/mL (Ertapenem/10 μg BIORAD was used as a positive control for *S. aureus,*(39) and fosfomycin/50 μg OXOID for *E. faecalis*(40) a filter paper disk with PBS only was used as a negative control), following the disk diffusion Kirby–Bauer method. Mueller–Hinton plates were incubated for 24 h at 37 °C.^41^ All treatments were carried out in triplicate. The normal distribution of data was confirmed by the Shapiro–Wilk test, and the group means were compared using a one-way analysis of variance (ANOVA) and Tukey’s test. The data were shown as the mean ± standard deviation (SD). The results were plotted on GraphPad Prism version 9.0. Differences between the variants were considered significant when *P* < 0.05.

### Characterizations

A Hitachi SU3500 scanning electron microscope was used to determine surface changes and chemical composition (MEB-EDS). A Bruker AXS D8 forward diffractometer operated at 35 kV and 25 mA was used to determine the zeolite crystal structure (XRD). A Bruker-Alpha tensor spectrophotometer was used to determine the molecular structure of the zeolite (FT-IR).

## Results and Discussion

**Figure 1:**
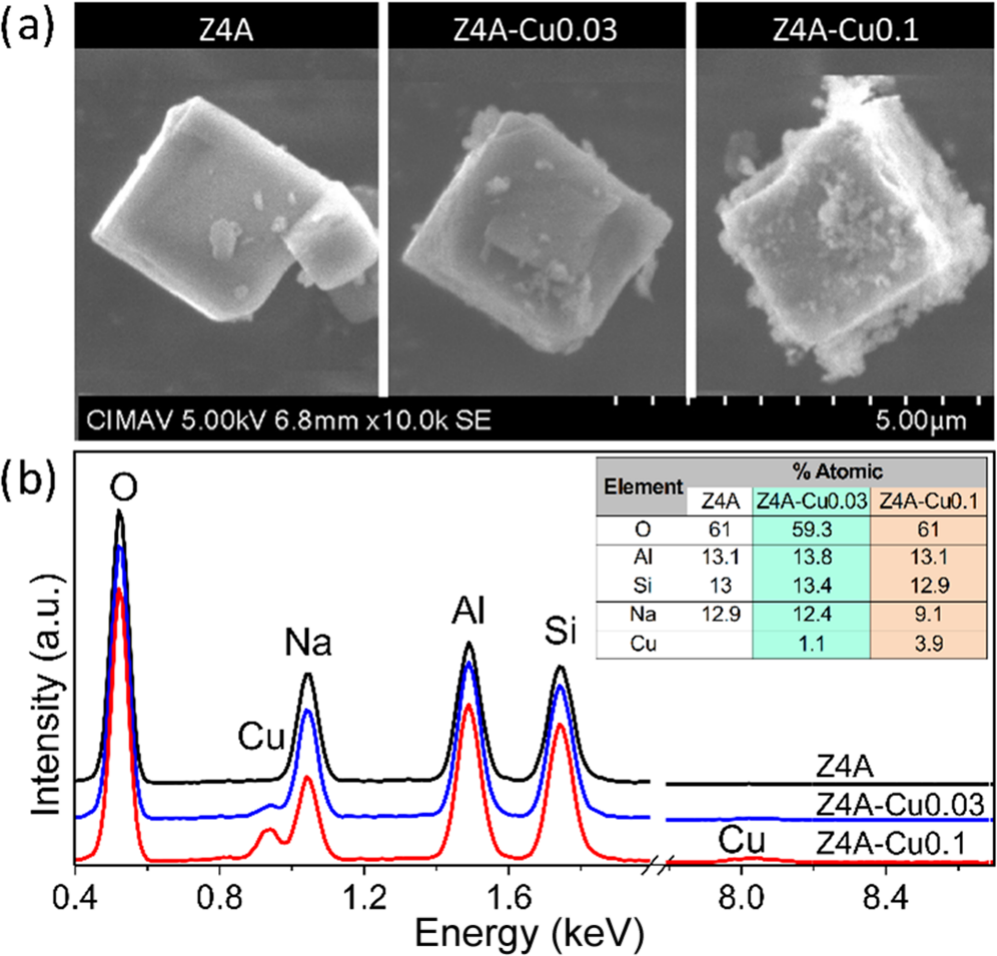
(a) SEM and (b) EDS analysis of Z4A, Z4A-Cu0.03, and Z4A-Cu0.1.

Figure 1 shows (a) SEM and (b) EDS analyses of Z4A, Z4A-Cu0.03, and Z4A-Cu0.1. In (a), a change in the surface of Z4A is observed with increasing Cu concentration regarding Z4A; this was caused by the chemical attack of the Cu solution. In (b), for Z4A, characteristic elements O, Na, Al, and Si with a 1:1:1 ratio (Na/Si/Al) are observed.

On the other hand, for Z4A-Cu0.03 and Z4A-Cu0.1, it is also observed that Cu at 0.93 keV has an evident increase related to its molar ratio (search for information about the ions in this region), conserving the 1:1:1 ratio (Na:Si:Al), which may indicate that the ionic exchange was performed in a satisfactory condition without excess Cu, which could cause agglomeration or particle formation.

**Figure 2:**
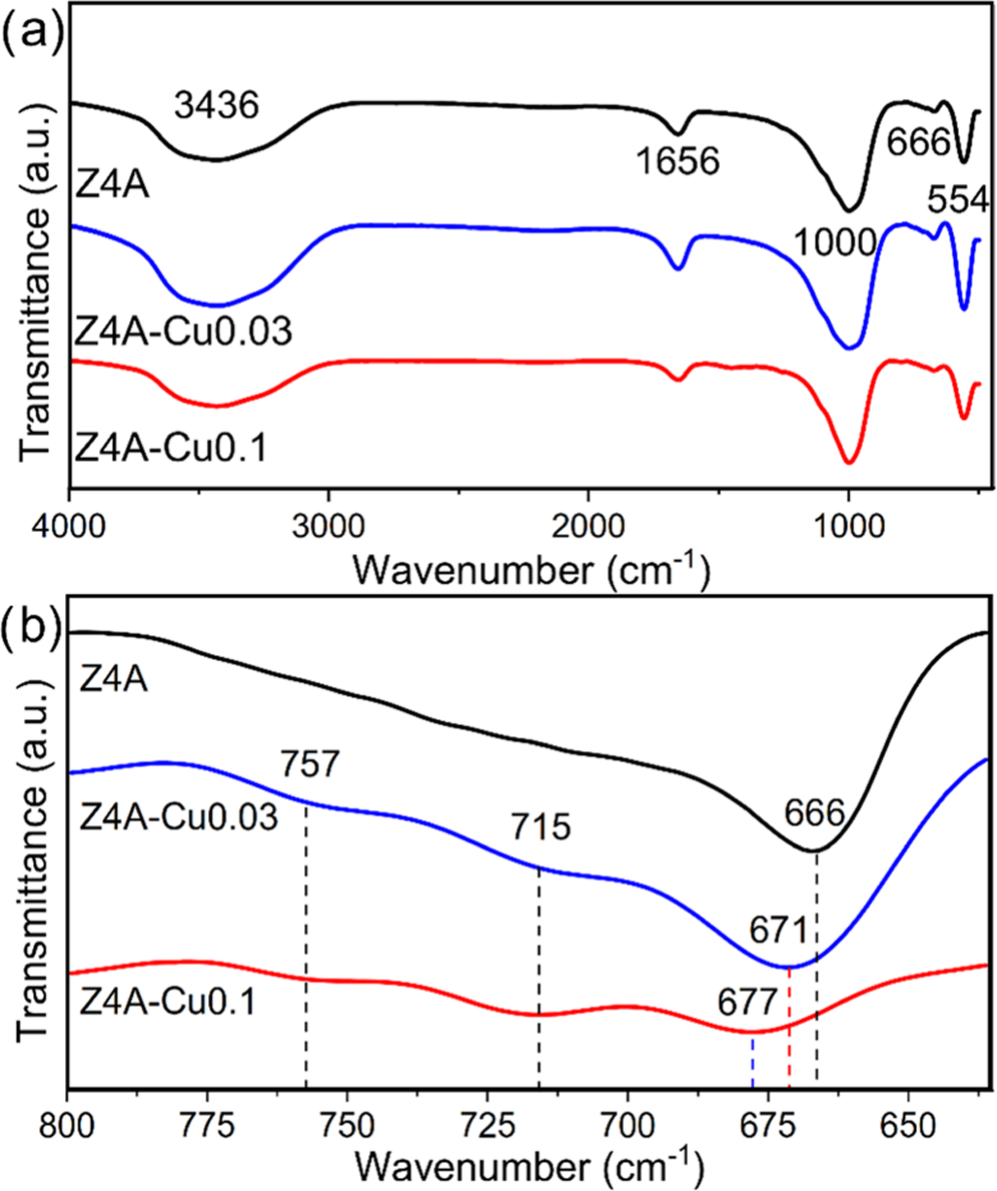
(a) FT-IR spectra and (b) approach in the 800–625 cm^–1^ region of Z4A, Z4A-Cu0.03, and Z4A-Cu0.1.

Figure 2 shows the FT-IR spectra of Z4A, Z4A-Cu0.03, and Z4A-Cu0.1. Figure 2a shows the band at 1000 cm^–1^, corresponding to the Si–O and Al–O asymmetric stretching vibration bonds (a characteristic region of the LTA zeolite). The peak at 666 cm^–1^ represents the Si–O–Al asymmetric stretching vibrational bond. The band at 554 cm^–1^ corresponds to the Si–O–Si and O–Si–O symmetrical stretching vibration bonds, attributed to the secondary structure (D4R); the bands at 1656 and 3436 cm^–1^ are assigned to the −OH vibrational stretching mode bonds of the water adsorbed on the zeolite.^8,42,43^ Similarly, in all spectra. Figure 2b shows the 666 cm^–1^ regions; in each spectrum (Z4A-Cu0.03 and Z4A-Cu0.1 regarding Z4A), novel bands can be observed in the range 675–780 cm^–1^. Moreover, an evident shift was observed in the copper samples regarding the 666 cm^–1^ band, which can be attributed to a disturbance in the chemical environment of Z4A due to the presence of ionic copper because this region is very sensitive to the changes associated with the interaction of Na^+^ and the Si–O–Al bonds. Therefore, an alteration in its molecular structure can lead to new interactions or band formation. However, the FT-IR region around 667 cm^–1^ has not been studied, and only an effect of similar behavior was found in the range close to 1000 cm^–1^, by the formation of CuO nanoparticles and copper polylsilicates in zeolite Y^44^ and LTA.^45^

**Figure 3:**
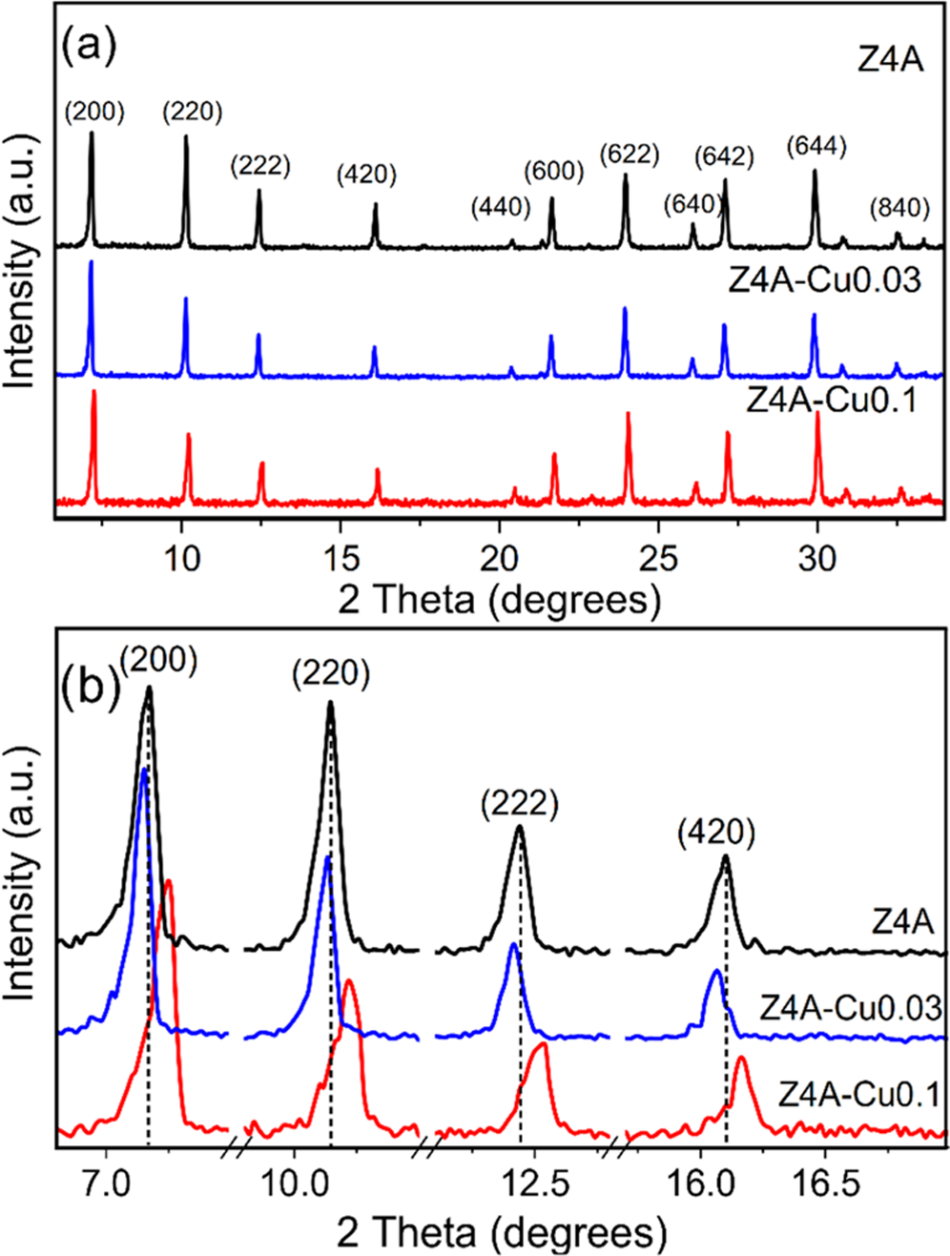
(a) X-ray diffraction and (b) approach to the highest peaks of Z4A, Z4A-Cu0.03, and Z4A-Cu0.1.

Figure 3 shows X-ray diffraction of Z4A, Z4A-Cu0.03, and Z4A-Cu0.1. Figure 3a shows the characteristic XRD patterns of Z4A, indexed by JCP2 01-073-2340, without any evidence of metallic particles or copper oxides. Figure 3b shows an enlargement of normalized X-ray diffraction peaks corresponding to peaks (200), (220), (222), and (420), where the behavior of Z4A is compared with Z4A-Cu0.03 and Z4A-Cu0.1. For Z4A-Cu0.1, there was a reduction in the relative intensity of peaks (220), (222), and (420) regarding Z4A and a shift toward higher angles in peaks (200), (220), (222), and (420), which causes stress in the crystalline structure of the zeolite.^29^ This could be produced by substituting one atom for another with a smaller radius, according to Bragg’s law, which indicates that the interplanar distance (*d*) is inversely proportional to the variation in degrees (2θ) and could be attributed to the substitution of Na^+^ (*r* = 95 pm) by Cu^2+^ (*r* = 72 pm).^46^ This may imply that, as the concentration of the Cu ion increases, the ion exchange reaction becomes more selective toward Cu^2+^ ions, which can be placed noncoplanar by substituting one Cu atom for two Na.^38,47^ For Z4A-Cu0.03 M, the relative intensity decreases in the (220), (222), and (420) peaks regarding Z4A, which can be attributed to the incorporation of Cu in Z4A. It can be observed that there is a displacement toward smaller angles in (200), (220), (222), and (420) peaks, which caused compression in the crystalline structure of the zeolite and is not an expected behavior if the incorporation of Cu were by Cu^2+^ ions. Additionally, we suppose that this effect is due to the incorporation of Cu^+^, which can be accommodated in a coplanar way by replacing a Cu atom with one of Na because the Na^+^ ion (*r* = 95 pm) when performing ion exchange with the Cu, which is estimated to be Cu^+^ (*r* = 96 pm)^48^ because the ionic radii of these are very similar. Analogous results have been reported for mono copper species identified as Cu^2+^, which demonstrate a direct interaction between the large cavity and the single 6-atom rings of zeolite.^27^

**Figure 4:**
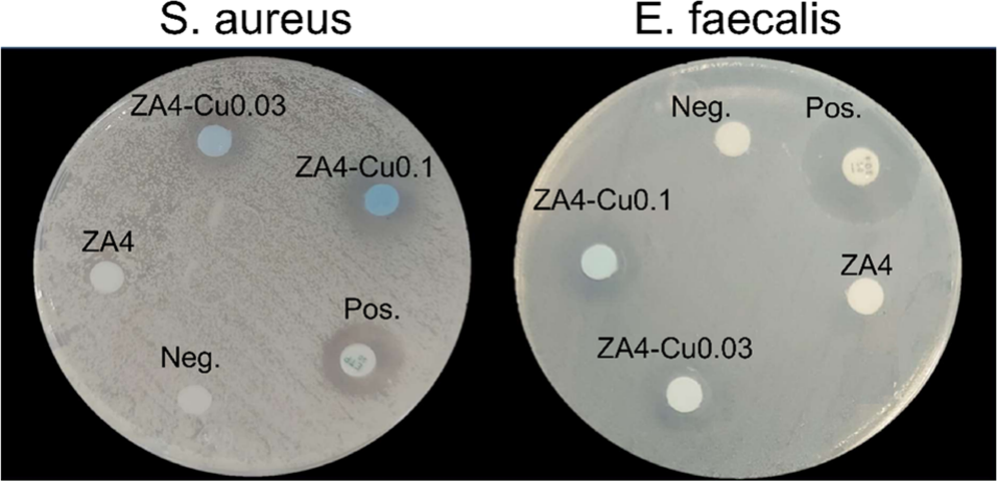
Diameter of the bacterial growth inhibition zones of Z4A, Z4A-Cu0.03, and Z4A-Cu0.1 against Gram-positive bacteria *S. aureus* and *E. faecalis*.

**Figure 5:**
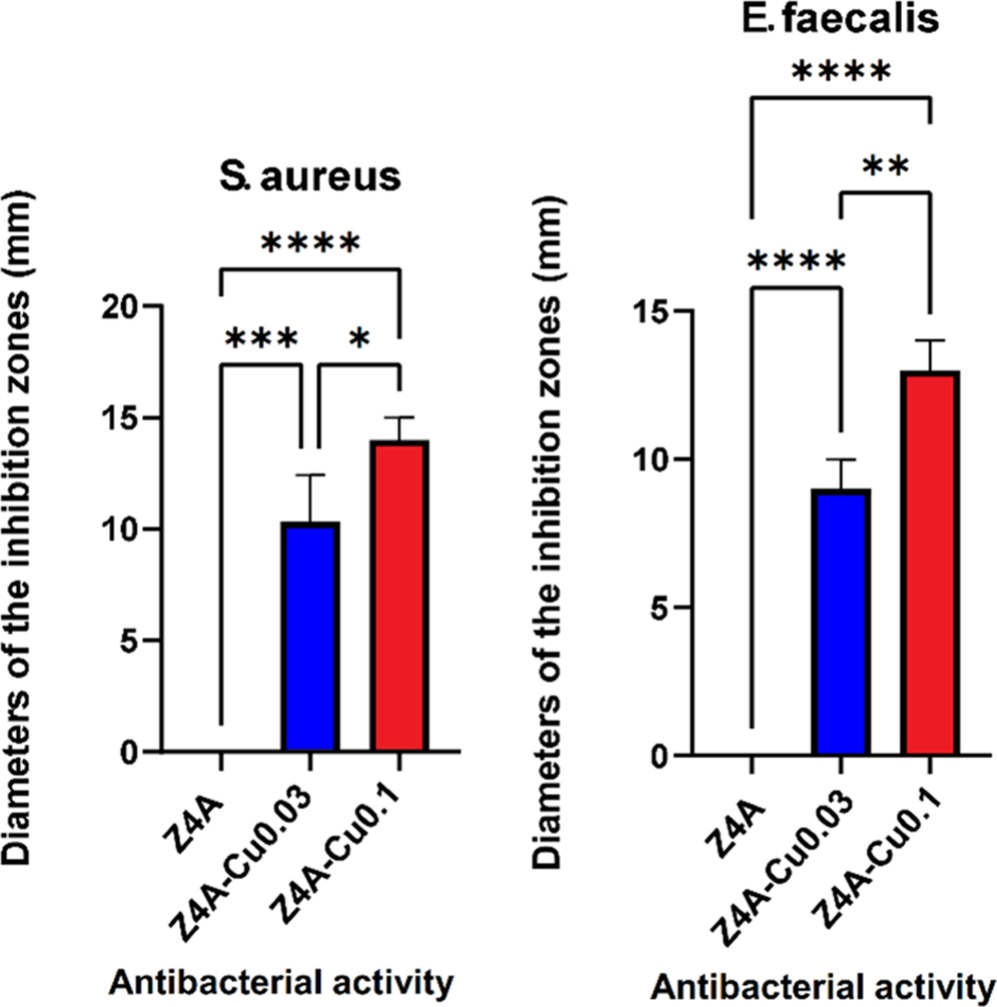
Graph of the antibacterial activity of Z4A, Z4A-Cu0.03, and Z4A-Cu0.1 against Gram-positive bacteria (left) *S. aureus* and (right) *E. faecalis*.

Figure 4 shows the diameter in mm of the bacterial growth inhibition zones of Z4A, Z4A-Cu0.03, and Z4A-Cu0.1 against Gram-positive bacteria *S. aureus* and *E. faecalis*. For Z4A, no bacterial inhibition halo is observed, as in the cases of Z4A-Cu0.03 and Z4A0.1. Figure 5 shows a graph of antibacterial activity; the results show a significant difference in bactericidal activity compared between Z4A-Cu0.03 and Z4A-Cu0.1, as well as no inhibition for Z4A. Table 1 shows the percentage inhibition of ZA4, ZA4-Cu0.03, and ZA4-Cu0.1 regarding the positive control for each of the bacterial strains tested. Comparing the effect of the materials versus the positive control on *S. aureus* growth, percent inhibition values of 67.4% and 91.3% for Z4A-Cu0.03 and Z4A-Cu0.1, respectively, show the bactericidal potential of the materials. Although in *E. faecalis*, the percentages of inhibition were lower compared to those observed for *S. aureus* (44.3% and 63.9% for Z4A-Cu0.03 and Z4A-Cu0.1, respectively), it is evident that there is a growth inhibitory effect on this bacterium.

**Table 1:** Inhibition Zone Size Around Antimicrobial Agents Tested by the Kirby–Bauer Disk Diffusion Method on Mueller–Hinton Agar^a^

		zone of inhibition (mm)		inhibition (%)	
material	dosis	*S. aureus*	*E. faecalis*	*S. aureus*	*E. faecalis*
Z4A	0.1 mg/mL	no zone	no zone	0	0
Z4A-Cu0.03	0.1 mg/mL	10.3 ± 2.1	9.0 ± 1.0	67.4 ± 0.9	44.3 ± 1.1
Z4A-Cu0.1	0.1 mg/mL	14.0 ± 1.0	13.0 ± 1.0	91.3 ± 1.3	63.9 ± 0.7
positive control	Ertapenem 10 μg	15.3 ± 0.6	N.A.		
	Fosfomycin 50 μg	N.A.	20.3 ± 0.6		

a N.A. = not applicable.

According to the antibacterial test results, the Z4A-Cu0.03 and Z4A-Cu0.1 materials affect *S. aureus* and *E. faecalis* in vitro; in both cases, the antibiogram test showed a growth inhibition zone. The results confirm the susceptibility of these strains to the materials, consistent with that reported in other studies.^49,50^ In addition, because the diameter of the inhibition halo is bigger invZ4ACu0.1 compared to Z4ACu0.03 for both (*S. aureus* and *E. faecalis*), XRD analysis allows us to elucidate that Z4ACu0.1 has been exchanged with Cu^2+^ ions and that Z4ACu0.03 has been exchanged with Cu^+^. Our results suggest that the inhibitory effect has a behavior dependent on the charge value of the Cu ion as well as on the copper concentration. The results of examining bacteria in the laboratory reveal a thorough understanding of how the pathogen and the antibacterial agent interact and how the properties of the materials are compared. This has been demonstrated in different studies.^51,52^

## Conclusions

The results suggested that ion exchange for Cu^2+^ occurred at a concentration of 0.1 M, where the relative intensity of the peaks decreased and there was displacement toward greater angles than in Z4A, which caused tension in its structure, leading to noncoplanar rearrangements due to the replacement of Na^+^ (*r* = 95 pm) by Cu^2+^ (*r* = 72 pm). The ion exchange for Cu^+^ occurred at a concentration of 0.03 M, at which the relative intensity of the peaks decreased and displacement toward smaller angles (2θ) than in Z4A was observed, which caused compression in its structure, accommodating in a coplanar way, due to the replacement of Na^+^ (*r* = 95 pm) by Cu^+^ (*r* = 96 pm). Furthermore, disturbance was observed in the ZA4 chemical environment, attributed to the presence of Cu^+^ and Cu^2+^ ions, which caused novel bands in the 667 cm^–1^ FT-IR region of the zeolite. Bactericidal tests showed good antibacterial activity mainly for ZA4-Cu0.1 (ion exchange selectivity for Cu^2+^), indicating that this material could be used as a first filter in the elimination of *S. aureus* and *E. faecalis* bacteria.
